# 

**Published:** 1879-09-20

**Authors:** 


### *EDITORIAL NOTICES.*

☞ *Explanation.*—The cross mark on our last Journal, intended as a reminder to subscribers in arrears, did not mean that the parties were all indebted for '78 and '79, but was aimed simply to cover both classes. Only a few seem to have noticed it. Friends, it was kindly meant; please respond to the call. You have no conception how much you trouble and incommode us by delay.—MANAGER.

*The Southern Medical College.*—This new institution will open on the Second Monday in October next.

Correspondents are requested to take notice that there are three medical schools in Atlanta:

THE SOUTHERN MEDICAL,
THE ATLANTA MEDICAL,
THE ECLECTIC.

Unless care be taken in the address, letters will fail to reach the parties intended. R. C. WORD, M.D., Dean pro tem.
*Southern Medical College.*

RECEIPTED.—1879 : T. L. Thomas ; A. J. Johnson ; Robert L. Tibbs ; Ezechiel Mann ; B. T. Jones ; Z. Hoffman ; Wm. Perrineau ; Foster L. Simpson ; Mathew Hopkins ; P. Pelham ; J. T. Jolly ; Patrick Malone ; Martin Fouche ; S. Masters : Eli Cates ; T. R. Lanier ; R. E. Toombs ; J. L. Lee ; J. E. Mount ; J. S. Horsely ; C. N. Howard ; L. M. Wood ; F. M. Fitzhugh ; J. W. Beckman ; D. Haygood ; O. M. Doyle. 1873 and '79 : W. D. Christy ; J. C. Tubb ; L. G. Lineeum.

